# Medical News and Miscellany

**Published:** 1895-12

**Authors:** 


### Medical News and Miscellany.

**Dr. W. T. Jones** has removed from San Marcos to Yoakum, Texas.

**Dr. S. F. Layton,** formerly of San Diego, Texas, is now at Alice, Texas.

**Dr. Malone Duggan** has removed from San Saba, Texas, to Eagle Pass.

**Dr. W. B. McKnight** has removed from Springtown, Texas, to Mansfield, Texas.

**Dr. T. W. Conerly,** has been appointed county physician of Tom Green county.

The *New York Medical Abstract* has suspended publication, with September number, Vol. XV., No. 9.

**Dr. T. J. Tyner** has been elected oculist to the school for the colored blind at Austin.

**Dr. B. A. Fowler,** of Brownwood, Texas, was married to Miss Mabel Looney of Hillsboro, on November 13th, ult.

**Philadelphia** has this year 2,610 medical students, against 2,345 last year; of these, 285 are homeopathic, and 200 are women.

**Married,** at Temple, Texas, Nov. 6, ult., Miss Wren Talley, daughter of Dr. R. P. Talley, of that city, to Mr. Leigh Hunt Hallam, of Dean, Texas.

**Dr. Ernest S. Lewis,** of New Orleans, was elected President of the Southern Surgical and Gynecological Association at its late meeting. He is Professor of Obstetrics in Tulane University, of which iustitution he is an alumnus of 1862.

**A physician** with first-class diploma and large reference can secure partnership in an excellent cash office business, by addressing Lock Box 275, Oklahoma City, Ok.

---

**Dr. Geo. J. Engelmann,** the celebrated surgeon-gynecologist of St. Louis, has removed to Boston, 336 Beacon Street. He has the Red-back's best wishes wherever he may be.

---

**Dr. F. S. White,** late superintendent of the State lunatic asylum at Austin, has removed to Terrell, purchased property and engaged in general practice. The doctor has the JOURNAL'S best wishes.

---

**A correspondent** taking occasion to thank the JOURNAL for its criticism on "Norma Trist, or Pure Carbon," suggests that sulphurated hydrogen would have been a more appropriate name for the story.

---

**Married.**—At the Presbyterian church, Honey Grove, Texas, November 12th, ult., Miss Jennie Jolley, daughter of Mr. and Mrs. J. F. Jolley, to Dr. E. E. Johnson, of Denison, Texas. The JOURNAL acknowledges the courtesy of a card.

---

**Dr. J. S. Steele,** of Hays county, Texas, is in Chicago studying the eye and ear in the great hospital clinic there, with the purpose of making the eye and ear a specialty. We commend the doctor to our Chicago friends as a gentleman worthy of their regard.

---

**For Sale.**—Our venerable friend, Dr. Paulus, recently deceased left a fine library. Among them is a set of eight volumes of Buck's Reference Hand-Book of Medical Science, as good as new, which will be sold for $35. Address D. A. Paulus, Hallettsville, Texas.

---

**Death of Dr. Conrad.**—Dr. H. B. Conrad, of New York, died in that city October 20, 1895. He was prominently connected with the various societies and teaching institutions of the city, being one of the faculty of the Medical Department of the University of the City of New York.

---

**Dr. A. S. Wolff,** the efficient and veteran quarantine officer at Brazos Santiago upon the close of the quarantine season was assigned to duty by State Health Officer Swearingen as special quarantine inspector and sanitary supervisor of the lower Rio Grande district with headquarters at Brownsville.

---

**Memphis Medical Monthly.**—A monthly Journal of Medicine and Surgery. Established 1880. Subscription price $1.00 a year. Commencing with the November issue, will publish, each month, liberally illustrated, one or two of the best *clinical* lectures delivered in the Memphis Hospital Medical College. Write for sample copy.

---

**Dr. W. M. Yandell**, of El Paso, and Dr. J. A. W. Wegefaith, have formed a copartnership for the general practice of medicine and Surgery, at El Paso. Dr. Wegefaith is an accomplished physician, formerly of Savannah, Georgia. He went to El Paso for the benefit of the climate, being predisposed to pulmonary disease. Dr. Yandell is a whole team within himself; together they will make a strong combination, and no doubt will do a splendid practice.

---

**The Tri-State.**—More space is devoted in this issue to the proceedings of the Tri-State Medical Society than we usually give to such matters, except the Texas State Medical Association. No apology is necessary, however, as the matter speaks for itself. Papers on very interesting subjects were read and discussed, and by men of national reputation, Westmoreland, Lindsey, Murfree, Davis and others, and the excellent Secretary, Dr. Frank Trester Smith, has gotten it up in a real chatty and attractive manner. It is good reading.

---

**Prof. Edw. Souchon,** of the Tulane University, has favored us with a reprint with illustrations, of his paper on "Drilling capillarary openings through the skull for exploring the brain with a needle and syringe," a process original with him, as a substitute for trephing in certain cases, a formidable operation. The paper was read before the Louisiana State Medical Society at a recent meeting. Prof. Souchon will send this reprint free on application. Prof. Souchon is investigating the operative treatment of irreducible dislocations of shoulder, recent or old, simple or complicated, and invites physicians who have operated cases to send him an account of them; also cases of persistent solidified aneurism, operated or not.

---

**Dr. E. T. Cook**, so well and favorably known in Texas, long time surgeon H. & T. C. R. R., has removed to Hot Springs

Ark., and formed a copartnership with Dr. W. H. Connell, of that city. Our loss is Arkansas' gain. We regret that the doctor found it necessary to make the change. It was on account of his wife's health. The climate of Houston did not agree with her. The doctor, in writing, expresses his regret at having to leave Houston, and says he never expects to find a better or a more congenial and clever people, or a better place to live in than Houston. Dr. Cook's old friends must not forget him when advising patients to go to the Springs.

---

We are requested by the Fort Worth Pharmacy Company, of Fort Worth, Texas, to say, that they will be pleased, upon application, to mail their Surgical Instrument Catalogue and Price List to any member of the profession.

Their catalogue quotes over six thousand articles and gives about the same number of cuts or figures of the articles.

They will also be pleased, at request, to mail special catalogues of Electric Batteries, Microscopes, Physicians' Chairs and Tables, and compressed air machines. which are now so successfully used by specialists in the treatment of throat and catarrhal diseases.

---

**Hamlet with the Prince Left Out.**—Under startling headlines, the *New York Medical Record* of October 5, announces "Calcification of an Orange Lying Free in the Pelvic Cavity. By Jno. A. Prince, M. D." As incredible as such thing seemed, we knew Prince to be good authority, so we read the article through several times. But we failed to find the slightest reference to an orange; the word "orange" does not appear in it, not even "the size of an orange." Dr. Prince told of a "uterus studded with fibroid nodules of small size," and says, "On passing my fingers over the posterior surface of the uterus they came in contact with a hard body, loosely adherent, and easily separated from its attachments to the uterus," but he didn't say it was an orange; didn't say what it was; as likely to have been a steamboat as an orange. But he says, "On examining the body (which, with the tube, was removed), it was found to be the exact size and shape of the ovary," which it was, of course. Where did Shrady get his toddies that day? It must have been "tanglefoot" or "cross-eye rye" variety.

---

**Fatal Mistake.**—Dr. W. H. Sutton, a leading physician of Dallas, injected three grains of strychnine in his arm by mistake for cocaine. He had for about nine years, it is said, been addicted to the use of morphine and cocaine hypodermically, and was in the habit of taking two or three grains of the latter at a dose, as occasion required. On this occasion—about 1:20 p. m., November 23rd—Dr. Martin, whose office adjoined Dr. Sutton's, hearing a noise in Dr. Sutton's room, entered. Dr. Sutton said, "Doctor, I have put three grains of strychnine in my arm by mistake for cocaine; if you can do anything for me, please do so quickly." Dr. Martin administered a little chloroform he had at hand, and ran to the drug store for assistance; on his return, Dr. Sutton was dead. These are the facts as given by the associated press dispatches.

Dr. Sutton was a native of Louisville, Ky., and a graduate of the Medical Department of the University of Louisville, of the class of 1862. He was 57 years of age, and had been practicing in Dallas about twenty-five years. Dr. Sutton was a very successful and much respected practitioner, and held in high regard by the regular medical profession. Dr. Sutton was married about eight months ago to his third wife. He was a member of the State Medical Association and of the Dallas County Medical Society.

---

**Linguistic Gymnastics.**—Elsewhere we have said that the students council of the Texas Medical College have begun the issue of a monthly periodical called the *University Medical*, to fill a long felt want of a vent to wind and wisdom. A writer in the first issue boils over with indignation at the recollection that the 24th Legislature curtailed the appropriation for the State University, and indulges in an extraordinary series of rhetorical ground and lofty tumbling, which is hard to beat. He takes a running start and begins thus:

"Before the gaze of the calm and dispassionate Texan who steps aside and critically examines the trend of current thought and action, weird and uncanny figures stand out in bold relief against the canvas of reason, dancing and reveling in wild orgies of supernal joy—skeleton priests who hold aloft the oriflamme and grinningly minister in the necropolis of educated thought."

Gee whiz!!

We submit, this demonstrates the need of an organ strongly put together at the joints, and justifies the venture.

The author then proceeds to set off a magnificent display of pyrotechnics, utterly demolishing the 24th Legislature, and leaving an ugly crack in the dome of the capitol,—(we had liked to say "creation"). He is particularly hard on "our politicians." He says, "our politicians strut like condescending Jupiters to the hustings with Northern hats on their heads."

We fancy it would be funny to see a condescending Jupiter, or any other kind of a Jupiter, on his way to the hustings or elsewhere—with a northern (with or without a big N) hat, or any other kind of a hat on his head. Or—was the hat on the head of the hustings? Steady there, young man!

---

**Dr. Frank Rainey,** of Austin, so long Superintendent of the State School for the Blind, has been appointed by Major-Gen. H. H. Boone, commanding the Department of Texas, Medical Director of the Department of Texas in the "United Confederate Veterans," and chief of his Medical Staff. This is a graceful compliment most worthily bestowed; but the beauty and appropriateness of the act can only be appreciated in the light of the following incident which occurred in the war times:

General Boone (then Major Boone) was in command of a squadron of cavalry on the Lafourche. Dr. Cupples, recently deceased, was Surgeon of Tom Green's Brigade, and Dr. Rainey Assistant Surgeon. In a desperate battle Boone was badly shot, and bled till his clothes were saturated. Drs. Cupples and Rainey amputated the Major's right arm and the fingers of the left hand, and when ready to dress the wound, could find no shirt to put on the wounded officer. It was then that Dr. Rainey showed his soldierly qualities and his bigness of heart. He stripped his own shirt from his back and put it on the wounded man, and as a consequence, caught such a cold that before day he was too hoarse to speak, and narrowly escaped pneumonia. The Major never knew anything about the shirt episode until he learned of it thirty odd years afterwards—at Houston last summer, during the Confederate reunion, on which occasion he was made Major-General of the United Confederate Veterans for the Department of Texas, vice General L. S. Ross.

The General says that a surgeon who will pull off his own shirt at midnight in midwinter and give it to a wounded comrade, deserves to be the Chief Surgeon of the layout,—and the JOURNAL thinks so, too. Bully for Boone and bully for Rainey.

---

### THE JOURNAL'S OWN PAGE.

Words of Cheer: Bro. Yandall, of El Paso, who contributes a paper on diphtheria in this number, and who, himself a veteran quill (having been president of the Texas Press Association), ought to know, writes: "The November number of the "Red Back" is the best number editorially that I remember to have seen of all the excellent editions since it was founded. You certainly did crucify the aspiring literary gent of La Grange." [On this subject the JOURNAL has received numerous letters of approval.]

---

"Couldn't well do without it.

"O. B. ATKINSON, Florence, Texas."

---

Dr. B. H. Rand, of Allen Farm, Texas, in renewing his subscription says:

"As this was my first year to subscribe for your journal, it is only fair for me to say that I am delighted with the 'Red-Back.' After having read it not quite one year, I am at a loss to understand how any doctor practicing in Texas can afford to be without it.

"With many thanks for your kindness, and best wishes for the 'Red-Back,' am very truly yours, B. H. RAND."

---

"Your journal is one of the best and most liked medical publications of the present day, and I can not be without it.

"H. WOLFF, M. D., Marlin, Texas."

---

Wanted—a young doctor, who is seeking a location, to travel over the State and take subscriptions for the Red Back. It takes like hot cakes, and as a very liberal arrangement will be made with the proper party, it can be made to pay well, and afford an opportunity to select an advantageous location. References imperatively required.

---

Delinquent subscribers are urged to settle up. If not convenient to pay in full, a remittance on account, as an evidence of good intentions, will be appreciated.

In response to our call, numbers have promptly remitted in full, some in part, for which "we 'uns" tender our most cordial thanks. We are publishing the most original, interesting and up to date journal anywhere; it is slap up, and we challenge any man to show anywhere more entertainment, instruction—"good value," as Moses says—for the sixteen cents than is found in the little "Red-Back" each month. "Push it along; it is a good thing."

---

"The JOURNAL is a publication I can't afford to do without Should I ever get in arrears draw on me and you will surely get your money." J. T. BENBROOK, M. D., Rockwall, Tex.

